# The Dynamic Field of Perioperative Treatment for Localized Muscle-Invasive Bladder Cancer: A Review of the Current Research Landscape

**DOI:** 10.3390/jcm14165653

**Published:** 2025-08-10

**Authors:** Clara García-Rayo, Silvia Juste-Álvarez, Carmen Gómez-Cañizo, Mario Hernández-Arroyo, Guillermo Velasco, Daniel Castellano, Alfredo Rodríguez-Antolín, Félix Guerrero-Ramos

**Affiliations:** 1 Department of Urology, Hospital Universitario 12 de Octubre, 28041 Madrid, Spain; clara.garciarayo@salud.madrid.org (C.G.-R.); silvia.juste@salud.madrid.org (S.J.-Á.); cgdelcanizo@salud.madrid.org (C.G.-C.); mario.hernandez@salud.madrid.org (M.H.-A.); alfredo.rodriguez@salud.madrid.org (A.R.-A.); 2 Department of Medical Oncology, Hospital Universitario 12 de Octubre, 28041 Madrid, Spain; gdvelasco.gdv@gmail.com (G.V.); cdanicas@hotmail.com (D.C.)

**Keywords:** urothelial cancer, muscle-invasive bladder cancer, radical cystectomy, immunotherapy, ctDNA, perioperative treatment, neoadjuvant therapy, adjuvant therapy

## Abstract

**Background**: Muscle-invasive bladder cancer (MIBC) is associated with high recurrence and mortality rates. While cisplatin-based neoadjuvant chemotherapy followed by radical cystectomy remains the standard of care, many patients are ineligible for cisplatin. Recent advances in immunotherapy and biomarker research are reshaping perioperative strategies, aiming to personalize treatment and improve outcomes. **Methods**: We conducted a comprehensive narrative review of the recent literature and clinical trials on the perioperative treatment of MIBC. We focused on published phase II and III trials assessing neoadjuvant and adjuvant strategies, including immunotherapy, antibody-drug conjugates (ADCs), combination regimens, and circulating tumor DNA (ctDNA)-based approaches. **Results**: Numerous trials (e.g., PURE-01, ABACUS, NABUCCO, AURA, NIAGARA) have demonstrated the feasibility and efficacy of immune checkpoint inhibitors (ICIs) in both cisplatin-eligible and -ineligible populations. Combination strategies, including ICIs plus chemotherapy or ADCs, have shown promising pathological complete response rates and event-free survival. In the adjuvant setting, nivolumab improved disease-free survival and received regulatory approval. Biomarkers such as PD-L1 and ctDNA are emerging tools for predicting treatment response and recurrence risk, although prospective validation is ongoing. **Conclusions**: The treatment paradigm for MIBC is shifting toward multimodal and biomarker-driven approaches. Integration of ICIs into perioperative management, especially in combination with chemotherapy or ADCs, may enhance outcomes. ctDNA shows potential as a predictive and prognostic biomarker, guiding therapeutic decisions and surveillance. Future research should focus on refining patient selection, optimizing treatment sequencing, and validating ctDNA-guided strategies to personalize care while minimizing overtreatment.

## 1. Introduction

Urothelial carcinoma (UC) of the bladder is the most prevalent malignancy of the urinary tract and poses a significant public health concern. Localized muscle-invasive bladder cancer (MIBC) represents 20–25% of all the new diagnoses of urothelial bladder cancer. Approximately 50% of patients undergoing radical cystectomy (RC) will experience disease recurrence or progression. In this context, neoadjuvant chemotherapy (NAC) with cisplatin-based regimens has been shown to improve overall survival (OS) in these patients and is considered the standard of care. However, despite its proven benefits, NAC remains underutilized in clinical practice [1,2].

**Figure 1:**
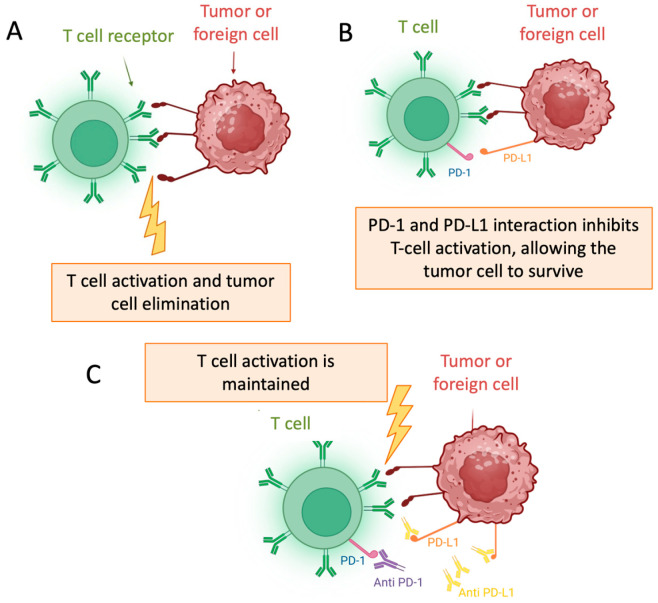
Mechanism of Action of Immune Checkpoint Inhibitors: (**A**) T cell activation; (**B**) PD-1/PD-L1 interaction; (**C**) Maintaned T cell activation [6].

In recent years, immunotherapy has transformed the standard of care for patients with locally advanced and metastatic UC, after the report of the results of JAVELIN, EV-302/KEYNOTE-A39, and CheckMate032 [3]. The success of immunotherapy in advanced settings has driven its development in the neoadjuvant and adjuvant scenarios. Emerging evidence from several phase 2 trials suggests that integrating immune checkpoint inhibitors (ICIs) with NAC may further enhance treatment outcomes in the perioperative setting. Notably, several phase 3 trials utilizing ICIs have reported results both with the adjuvant and perioperative approach [1,4]. These findings reflect a growing shift in interest towards immunotherapy-based approaches in the perioperative management of MIBC, whose mechanism of action is shown in Figure 1.

However, localized MIBC is still a therapeutic challenge due to high recurrence rates and significant mortality, underscoring the need for further research to optimize treatment strategies. In parallel with the development of novel therapies, growing attention is also being directed toward the personalization of treatment. Biomarker-driven approaches are becoming central to identifying the patients most likely to benefit from specific interventions. Among the emerging tools, circulating tumor DNA (ctDNA) is currently the most promising biomarker for treatment response assessment, minimal residual disease detection, and early relapse prediction. Its integration into perioperative trials may help refine treatment and patient selection and monitoring.

This review aims to provide an update on the perioperative management of MIBC, focusing on the most recent data and giving an overview of the current clinical trial landscape, which will lead to new developments in the near future [5].

## 2. Materials and Methods

This narrative review was conducted to provide a comprehensive and up-to-date synthesis of the evolving perioperative treatment landscape for MIBC. A systematic search of the literature was performed using the PubMed/MEDLINE, Embase, and ClinicalTrials.gov databases to identify relevant publications from January 2015 to April 2025. The search strategy combined medical subject headings (MeSH) and free-text terms including “muscle-invasive bladder cancer,” “urothelial carcinoma,” “neoadjuvant therapy,” “adjuvant therapy,” “immunotherapy,” “chemotherapy,” “antibody-drug conjugates,” and “circulating tumor DNA”.

Only English-language articles focusing on non-metastatic MIBC were considered. Priority was given to phase II and III clinical trials, systematic reviews, meta-analyses, and international guidelines. Abstracts from major Urology and Oncology conferences—such as those organized by ASCO, ESMO, EAU, and SUO—were also reviewed to capture the most recent unpublished data. Reference lists of key studies were manually screened to identify additional relevant publications.

Data extraction focused on study design, population characteristics, treatment regimens, clinical endpoints (e.g., pathological complete response (pCR), event-free survival (EFS), disease-free survival (DFS)), and biomarker integration). Special emphasis was placed on emerging therapeutic strategies, including immune checkpoint inhibitors, antibody-drug conjugates (ADCs), and ctDNA-guided interventions. The included evidence was then categorized and synthesized by therapeutic setting (neoadjuvant, adjuvant, or perioperative) and patient eligibility (cisplatin-eligible vs. cisplatin-ineligible), with the aim of outlining current standards and ongoing investigational efforts in this rapidly evolving field.

## 3. Synthesis of the Evidence

### 3.1. Neoadjuvant Treatment

The VESPER trial (GETUG-AFU V05) compared the efficacy of dose-dense methotrexate, vinblastine, doxorubicin, and cisplatin (dd-MVAC) versus gemcitabine–cisplatin (GC) as NAC. Results showed that dd-MVAC improved 3-year progression-free survival (PFS) compared to GC in the neoadjuvant setting, although no significant difference in OS was observed in the overall study population. Additionally, a higher rate of pCR (pT0) was reported in the dd-MVAC group [7]. Nevertheless, no standardized perioperative approach has yet been established for cisplatin-ineligible patients.

In the last few years, numerous clinical trials have explored the role of immunotherapy, ADCs, and novel delivery systems in the neoadjuvant setting for MIBC. The primary goals of these studies are to enhance pathological response rates, improve survival outcomes, and eventually lead to bladder preservation in both cisplatin-ineligible and eligible patients.

#### 3.1.1. Neoadjuvant Single-Agent Immunotherapy and Antibody Drug Conjugates

PURE-01 (pembrolizumab) and ABACUS (atezolizumab) are two completed phase II trials with their final results published. PURE-01 evaluated pembrolizumab monotherapy in cisplatin-eligible patients, reporting a pCR rate of 42%, with 3-year EFS and OS rates of 74.4% and 83.8%, respectively. This study highlighted the potential of pembrolizumab to induce significant tumor regression preoperatively, particularly in patients with high programmed death-ligand 1 (PD-L1) expression or high tumor mutational burden (TMB) [8]. ABACUS tested atezolizumab in the same population, achieving a pCR rate of 31% with 2-year recurrence-free survival (RFS) and OS rates of 77% and 82%, respectively. This trial also explored biomarkers such as PD-L1 expression and ctDNA as potential predictors of response and long-term outcomes [9]. Both trials demonstrated the safety and feasibility of immunotherapy as a neoadjuvant approach and justified further trials with these and other different drugs, as shown in Table 1.

Additionally, the ABACUS-2 trial investigated the role of neoadjuvant atezolizumab in histological subtypes of bladder cancer, showing higher efficacy in sarcomatoid UC, as compared with other histologies. However, it also highlighted the inconsistency in the histopathological classification of these subtypes, posing significant challenges to the development and evaluation of studies targeting this subgroup [10].

Novel agents such as ADCs are being explored. These agents have the potential to induce immunogenic tumor cell death, promoting the activation and recruitment of immune cells. Sacituzumab govitecan targets trophoblast cell-surface antigen 2 (TROP2), a protein that promotes tumor cell proliferation and is frequently overexpressed in UC. It has gained U.S. FDA-accelerated approval for the treatment of locally advanced or metastatic UC based on the results of TROPHY-U-01 [11,12]. The SURE-01 trial explored the role of neoadjuvant immunotherapy using sacituzumab govitecan in cisplatin-ineligible patients. This phase II, single-arm study demonstrated a pCR rate of 36.8% and pathological downstaging (≤pT1N0) in 57.9% of evaluable patients. The treatment demonstrated grade 3 or higher treatment-related adverse events rates of 52.4%. These results suggest that sacituzumab govitecan, traditionally used in metastatic settings, may also be effective in earlier stages when used as neoadjuvant monotherapy, offering a promising alternative for patients not suitable for platinum-based chemotherapy [13].

#### 3.1.2. Neoadjuvant Immunotherapy Combination

As a result of the positive outcomes of immunotherapy in cancer treatment, other treatment alternatives are being explored, such as combinations of different immunotherapeutic agents, as shown in Table 2.

The phase Ib NABUCCO trial investigated the feasibility and efficacy of neoadjuvant immunotherapy combining ipilimumab (anti-cytotoxic T-lymphocyte–associated protein 4 (CTLA-4)) and nivolumab (anti-programmed death-1 (PD-1)) in patients with stage III UC. In Cohort 1, 24 patients received two doses of ipilimumab at 3 mg/kg and two doses of nivolumab at 1 mg/kg, resulting in a pCR rate of 46%. To optimize the dosing regimen, Cohort 2 enrolled 30 additional patients randomized to two arms: Cohort 2A received ipilimumab 3 mg/kg plus nivolumab 1 mg/kg, while Cohort 2B received ipilimumab 1 mg/kg plus nivolumab 3 mg/kg. The pCR rates were 43% in Cohort 2A and 7% in Cohort 2B, indicating superior efficacy with higher ipilimumab dosing. Additionally, the absence of ctDNA in plasma prior to surgery correlated with higher pCR rates and improved PFS, suggesting its potential as a predictive biomarker [14].

There are other studies currently exploring the combination of ICIs, like NCT02845323, a phase II clinical trial evaluating the post cystectomy CD8+ tumor response of patients receiving nivolumab plus urelumab versus nivolumab alone, whose results will soon be available.

Another innovative approach under investigation is the combination of ICIs with agents targeting DNA repair mechanisms. The NEODURVARIB trial is a phase II study evaluating the combination of durvalumab and olaparib, a poly (ADP-ribose) polymerase (PARP) inhibitor, in a neoadjuvant fashion for patients with localized MIBC. Of the 29 patients treated, a pCR was observed in 13 cases (44.8%) and 26 patients (90%) underwent RC. While genomic alterations remained stable throughout treatment, transcriptomic analysis revealed that resistant tumors were enriched in epithelial–mesenchymal transition and transforming growth factor beta (TGF-β) signatures, along with a luminal-to-basal phenotype shift. Increased PD-L1 expression and the presence of circulating senescent T cells were associated with pCR, suggesting potential biomarkers of treatment response and resistance [15].

A different trial investigated a novel oncolytic immunotherapy approach combining intravesical cretostimogene grenadenorepvec (also known as CG0070)—a genetically modified adenovirus encoding granulocyte–macrophage colony-stimulating factor—with systemic nivolumab in cisplatin-ineligible patients with cT2–T4aN0–1M0 MIBC. In this phase Ib study, the combination was well tolerated with no dose-limiting toxicities in 21 treated patients. The regimen achieved a pCR rate of 42.1% and 1-year RFS of 70.4%. Notably, complete response correlated with a high TMB and baseline free E2F activity, rather than PD-L1 status. Importantly, the presence and maturation of tertiary lymphoid structures post-treatment were strongly associated with response, underscoring the relevance of both humoral and cellular immune activation [16].

#### 3.1.3. Neoadjuvant Combination of Immunotherapy and Chemotherapy

Several ongoing clinical trials are evaluating the integration of ICIs into NAC regimens, aiming to enhance oncologic outcomes beyond those achieved with chemotherapy alone, as shown in Table 3.

The AURA trial is a multicenter, randomized, non-comparative phase II study evaluating the efficacy and safety of neoadjuvant avelumab, alone or in combination with chemotherapy. Patients were stratified based on cisplatin eligibility. In the cisplatin-eligible cohort, patients received either GC plus avelumab or dd-MVAC plus avelumab. The pCR rates were 54% and 58%, respectively, with 36-month OS rates of 64% and 85%, respectively. In the cisplatin-ineligible cohort, patients received either paclitaxel/gemcitabine plus avelumab or avelumab monotherapy. The pCR rates were 14% and 33%, respectively, with 12-month OS rates of 67% and 75% [17].

TAR-200 is an intravesical drug delivery system designed to provide sustained local release of gemcitabine directly within the bladder [18]. Initially developed for non-muscle-invasive bladder cancer (NMIBC), TAR-200 has shown promising results in patients unresponsive to Bacillus Calmette–Guérin (BCG) therapy [19,20]. Building upon these findings, the SunRISe-4 trial extended the application of TAR-200 to the neoadjuvant setting for MIBC. In this phase II study, cisplatin-ineligible or -refusing patients with MIBC received either intravesical TAR-200 combined with the systemic PD-1 inhibitor cetrelimab or cetrelimab alone prior to RC. Interim analysis revealed that the combination therapy achieved a pCR rate of 42%, compared to 23% with cetrelimab monotherapy. Notably, in patients with organ-confined disease (cT2), the pCR rate was 48% with the combination therapy [21,22].

In addition to the development of novel therapeutic agents, advances in prognostic modelling are being explored to optimize treatment strategies in MIBC. A recent study applied artificial intelligence (AI) to analyze nuclear morphology features from pre-treatment transurethral resection of bladder tumor (TURBT) specimens in the BLASST-1 trial, which evaluated neoadjuvant nivolumab combined with GC. Utilizing machine learning models, researchers extracted 408 features related to nuclear texture and spatial arrangement. The 17 most significant features were used to train a Cox regression model, which demonstrated prognostic capability in The Cancer Genome Atlas dataset and predictive accuracy for pathological downstaging in the BLASST-1 cohort, with an area under the Receiver Operating Characteristic curve of 0.83 [23].

### 3.2. Adyuvant Treatment

#### 3.2.1. Adjuvant Platinum-Based Chemotherapy

There is limited high-quality evidence supporting routine adjuvant chemotherapy (AC) after RC in high-risk patients, due to methodological flaws in existing randomized controlled trials (RCTs). Patients should be informed of the limited data and potential AC regimens, including CMV, CISCA, MVAC, CM, cisplatin monotherapy, and modern combinations like GC [24].

Kronstedt et al. reported in a meta-analysis (n > 2400 patients) that early AC (within 45 days post-RC) significantly improves OS and PFS compared to delayed AC, emphasizing the importance of timely administration [25].

#### 3.2.2. Adjuvant Single-Agent Immunotherapy

Three phase III RCTs have investigated the efficacy of PD-1/PD-L1 checkpoint inhibitors in patients with MIBC, muscle-invasive UC (MIUC) of the ureter and renal pelvis at high risk of recurrence, as summarized in Table 4. Although both the AMBASSADOR and CheckMate 274 trials reported a benefit in DFS, only CheckMate 274 demonstrated a positive trend in OS based on Kaplan–Meier curves, as the necessary follow-up duration to assess long-term outcomes has not yet been reached [26]. Based on these findings, the FDA has approved adjuvant nivolumab for patients with high-risk MIUC post-surgery, while the EMA has approved it for adults with MIUC expressing PD-L1 ≥ 1%, who are at high risk of recurrence after radical resection [27,28].

Updated results from CheckMate 274, with a median follow-up of three years, confirmed a sustained 15% improvement in DFS with adjuvant nivolumab. An OS benefit of 11% was observed in patients with PD-L1 expression ≥ 1%. These findings reinforce adjuvant nivolumab as a standard of care for high-risk MIUC with potential curative intent [26]. Additionally, a subcutaneous formulation of nivolumab has demonstrated comparable efficacy to intravenous administration, offering a more convenient alternative for both patients and physicians.

A post-hoc analysis of IMvigor010 [29] highlighted ctDNA as a potential prognostic and predictive biomarker in the adjuvant setting after atezolizumab. ctDNA was detectable in 37% of patients and was associated with poorer DFS and OS. In ctDNA-positive patients, atezolizumab significantly improved both outcomes, emphasizing ctDNA’s utility in identifying patients most likely to benefit from adjuvant immunotherapy. ctDNA clearance after two cycles correlated with better OS, and serial ctDNA testing predicted relapse more accurately than baseline testing alone, with a lead time of approximately 3.8 months. However, 32% of ctDNA-negative patients still relapsed, indicating limitations. In this context, the IMvigor011 trial is ongoing to evaluate ctDNA-guided adjuvant strategies [30].

The AMBASSADOR trial supports pembrolizumab as a promising addition to the therapeutic landscape for high-risk MIUC, particularly for cisplatin-ineligible patients. The substantial DFS benefit observed could justify its integration into clinical practice. However, the lack of mature OS data—partly due to substantial crossover from placebo to adjuvant nivolumab following FDA approval—warrants cautious interpretation, suggesting that the widespread adoption of pembrolizumab should be considered within a broader multidisciplinary framework [31].

Following IMvigor010 demonstration of ctDNA’s predictive value, several ongoing trials are investigating which MIBC patients benefit most from adjuvant immunotherapy, highlighting the importance of personalized treatment strategies [32], as described in Table 5.

IMvigor011 uses postoperative ctDNA status to guide adjuvant treatment. It randomizes only ctDNA-positive patients to receive adjuvant atezolizumab versus observation, but monitors all subjects (both ctDNA-positive and negative) with serial ctDNA assessments. Early results in ctDNA-negative patients (n = 171) show high 12- and 18-month DFS rates (92% and 88%, respectively) and OS rates (100% and 98%, respectively), supporting surveillance in this subgroup. This trial advances personalized adjuvant therapy in high-risk MIBC by using ctDNA as a marker of minimal residual disease, aiming to minimize unnecessary treatment exposure in ctDNA-negative patients [33,34].

Furthermore, the TOMBOLA study, a non-randomized phase III trial, also investigated a ctDNA-guided intervention strategy [35]. In contrast to the IMvigor011 trial, the TOMBOLA study administers atezolizumab to all ctDNA-positive patients. Notably, 56% of patients were ctDNA-positive post-cystectomy. Among ctDNA-negative patients, only 3% developed metastases—a relapse rate lower than that observed in IMvigor011 (which reported a 10% metastasis rate in ctDNA-negative patients). Among ctDNA-positive patients, 55% achieved ctDNA negativity and radiographic disease-free status following atezolizumab therapy. Overall, recurrence-free survival one year after RC was excellent in ctDNA-negative patients (98.5%) compared to ctDNA-positive patients (75.2%) (*p* < 0.001). These findings suggest that ctDNA-directed interventions may offer meaningful survival benefits in the adjuvant setting.

Efforts to address the limitations of CheckMate 274—particularly its inability to clearly identify which patients do not benefit from adjuvant therapy—have led to the development of the MODERN trial (NCT05987241). This randomized phase II/III study evaluates ctDNA-guided adjuvant treatment in MIBC, as described in Table 5. By integrating dynamic biomarkers such as ctDNA, the MODERN trial aims to refine strategies for both treatment escalation and de-escalation based on molecular residual disease status [36,37].

### 3.3. Perioperative Treatment

The perioperative treatment landscape for MIBC has become an area of increased interest, historically dominated by cisplatin-based chemotherapy regimens. However, for cisplatin-ineligible patients, no standardized perioperative approach has yet been established. Emerging data from immunotherapy-based regimens and combination strategies are redefining treatment paradigms in both groups.

#### 3.3.1. Perioperative Trials for Cisplatin-Eligible Patients

In Table 6, we summarize the features of the main clinical trials that investigate the use of immunotherapy in a perioperative management, as a “sandwich” approach, with standard NAC with GC for cisplatin-eligible patients.

The NIAGARA trial recently demonstrated a significant benefit in both co-primary endpoints (OS and EFS). The pCR rate with NAC plus perioperative durvalumab was 33.8% vs. 25.8% with NAC alone. EFS at 2 years was 67.8% in the durvalumab arm vs. 59.8% in the control arm (HR 0.68). The 24-month OS rate was 82.2% in the durvalumab group vs. 75.2% in the control group (HR for death 0.75). Among other secondary endpoints, the risk of MFS was reduced by 33%, and the risk of a DSS event was reduced by 31%. No new safety signals were observed with the use of durvalumab. Recent findings also suggest that ctDNA clearance after NAC is associated with improved EFS. The addition of durvalumab to NAC increased ctDNA clearance by 13% [38,39]. These results have led to approval by the European Medicines Agency (EMA) in July 2025.

To date, no results have been published yet from the KEYNOTE-866 trial.

PD-L1 expression increases in MIBC post-NAC, supporting a combined PD-1/PD-L1 blockade. Additionally, indoleamine 2,3-dioxygenase 1 (IDO1) is highly expressed in bladder cancer and is linked to poor prognosis. Linrodostat mesylate (BMS-986205), a potent, selective oral inhibitor of IDO1, has demonstrated an acceptable safety profile and preliminary signs of clinical efficacy when combined with nivolumab in metastatic UC (37% objective response rate in treatment-naïve patients). Within this framework, the ENERGIZE trial, launched in 2018, assesses the role of NAC + nivolumab + linrodostat [40].

#### 3.3.2. Perioperative Trials for Cisplatin-Ineligible Patients

##### Combination of Chemotherapy and Immunotherapy

The NURE-Combo phase II trial was the first to demonstrate the efficacy of neoadjuvant nivolumab plus nab-paclitaxel followed by adjuvant nivolumab in cisplatin-ineligible MIBC patients (cT2–4aN0–1M0) with predominant urothelial histology. The pCR rate was 39%, and 73.3% achieved ≤ypT1N0. The 12-month EFS rate was 89%. Biomarker analyses indicated certain myeloid cell populations as potential predictors of pCR.

##### Combination of Immunotherapy and ADC

There are several ongoing clinical trials combining immunotherapy with ADCs in the neoadjuvant setting. However, none of these trials have reported results to date. The EV-303/KEYNOTE-905 trial (NCT03924895) is a multicenter, randomized, open-label, phase III study evaluating the efficacy and safety of perioperative pembrolizumab alone or combined with enfortumab vedotin (EV) versus radical cystectomy alone in patients with MIBC who are ineligible for, or decline cisplatin-based treatment [41]. This approach is supported by the positive results of the EV-302/KEYNOTE-A39 trial (NCT04223856), which recently established this combination as the new standard of care in the first-line treatment of metastatic urothelial carcinoma [3].

EV is also being investigated in combination with other immunotherapeutic agents in the perioperative setting. The phase III VOLGA trial (NCT04960709) is evaluating the efficacy and safety of perioperative durvalumab plus EV, with or without tremelimumab, in cisplatin-ineligible individuals. This study aims to determine whether these combinations can improve pCR rates and EFS compared to standard of care [42].

Beyond EV, other ADCs are being evaluated in the neoadjuvant setting, including HER2-directed therapies. The phase II RC48-C017 trial (NCT05297552) investigates the combination of disitamab vedotin (DV), an anti-HER2 ADC, with the PD-1 inhibitor toripalimab in patients with HER2-expressing (IHC ≥1+) MIBC. The most recent results showed a pCR rate of 63.6% among patients who underwent radical cystectomy. The combination was also well tolerated, with most adverse events being grade 1–2 and no surgical delays attributed to treatment [43].

In addition to the evaluation of sacituzumab govitecan as monotherapy (SURE-01 trial), this compound is also being studied in combination with pembrolizumab in the perioperative setting. The SURE-02 trial (NCT05239728) is a phase II study assessing the safety and efficacy of this combination in patients with MIBC who are ineligible for cisplatin. This study included 36 patients, showing encouraging efficacy, with a pCR rate of 44% and a pathological downstaging (≤pT1N0) rate of 55%, allowing bladder preservation without chemo-radiotherapy in 74% of patients refusing RC. Treatment was generally well tolerated, with no new safety signals [44].

A variety of novel agents are being investigated in the perioperative setting. One of these agents is bempegaldesleukin (NKTR-214), an engineered interleukin-2 (IL-2) cytokine prodrug designed to selectively stimulate the IL-2 receptor βγ complex, promoting CD8^+^ T cell and NK cell proliferation while minimizing regulatory T cell activation. The phase III PIVOT IO 009 trial (NCT04209114) evaluates the combination of nivolumab with bempegaldesleukin versus nivolumab alone or standard of care in cisplatin-ineligible patients. However, the global clinical development of bempegaldesleukin was discontinued following disappointing efficacy results in the studies, and the PIVOT IO 009 trial was terminated early without publication of the results [45,46] (Table 7).

## 4. Discussion

**Figure 2:**
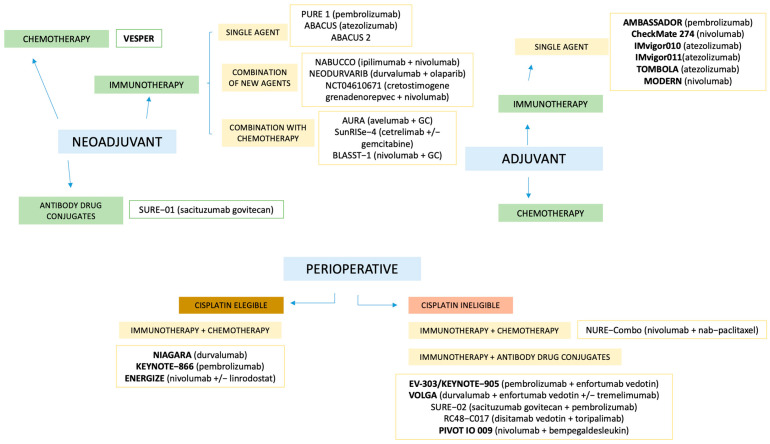
Summary of available studies across different clinical scenarios. Studies in bold represent Phase III trials. GC: gemcitabine + cisplatin.

The treatment landscape of MIBC is rapidly evolving beyond platinum-based chemotherapy, driven by the need to improve oncologic outcomes and expand options for cisplatin-ineligible patients, as illustrated in Figure 2, which provides a summary of the available studies in the different scenarios.

In the neoadjuvant scenario, the most influential early-phase trials, PURE-01, conducted in cisplatin-eligible patients, and ABACUS, which enrolled cisplatin-ineligible individuals, demonstrated promising pCR rates of 42% and 31%, respectively, using pembrolizumab and atezolizumab monotherapy [9]. While both studies laid the foundation for ICI in the neoadjuvant setting, their non-randomized designs, modest sample sizes, and relatively short follow-up limit the generalizability of findings.

It is widely recognized that histological subtype is a relevant determinant of response, with variant histologies generally associated with poorer outcomes. Several studies are currently addressing the challenge of treating these specific subpopulations, such as the ABACUS 2 trial, which explored the use of neoadjuvant atezolizumab in different histological subtypes, showing greater drug sensitivity in tumors with a sarcomatoid variant. This and other post-hoc studies have highlighted the need for a consistent histological classification and dedicated trials in variant histologies [9,10,11].

Furthermore, combination approaches—whether with chemotherapy (e.g., AURA), dual ICIs (e.g., NABUCCO), or novel agents like ADCs or oncolytic viruses—aim to overcome primary resistance and broaden treatment applicability [17,47]. However, many of these remain in early development or lack comparative data against standard chemotherapy.

In the adjuvant approach, CheckMate 274, AMBASSADOR and IMvigor010 have investigated the efficacy of ICIs. CheckMate 274, which evaluated adjuvant nivolumab, reported positive results for both OS (immature) and DFS, while AMBASSADOR, investigating pembrolizumab, reported positive results only for DFS; and IMvigor, using atezolizumab, failed to meet its primary endpoints. One hypothesis is that anti-PD-1 agents like nivolumab and pembrolizumab may more effectively engage T cells to elicit a stronger immune response compared to anti-PD-L1 agents [48]. However, these discrepancies likely reflect differences in trial design, patient selection criteria—such as the number of patients randomized with upper tract urothelial carcinoma—and the pharmacodynamics of PD-1 versus PD-L1 inhibitors. These factors make direct cross-trial comparisons challenging, which should always be approached with caution. While DFS is often used as a surrogate for OS in adjuvant trials, its reliability in predicting long-term outcomes in MIUC remains unclear. For some cancers, such as breast or colorectal cancer, DFS closely correlates with OS, but this relationship is less established in UC [49]. One possible explanation for the lack of OS advantage from pembrolizumab in AMBASSADOR is the substantial rate of crossover and censoring following consent withdrawal: 58/348 (17%) patients in the observation group and 46/354 (13%) patients in the pembrolizumab group either withdrew consent or received another ICI before progression. Additionally, among patients who experienced disease progression, 22 (15%) in the pembrolizumab group and 83 (52%) in the observation group received subsequent treatment with an ICI. With a 45-month median follow-up, OS data may still mature, but the rate of crossover complicates future analyses on OS.

Regarding perioperative management, the positive results of NIAGARA support perioperative durvalumab with neoadjuvant chemotherapy as a potential new standard of care for patients with cisplatin-eligible MIBC, and this has been translated into EMA approval. However, a perioperative approach remains an open question between a universal upfront treatment approach versus a risk-adapted strategy. Critics highlight that some patients achieving a pCR after neoadjuvant therapy may derive minimal additional benefit from continued adjuvant immunotherapy, raising the risk of overtreatment [36]. Although grade 3–4 adverse events were similar between study arms (~41%) and without notable surgical delays, immune-related toxicities and the expense of prolonged adjuvant therapy warrant evaluation, particularly for patients with minimal residual disease [39].

In addition to the investigation of novel therapeutic agents, significant efforts are underway to personalize perioperative treatment strategies for MIBC, allowing clinicians to identify patients most likely to benefit while avoiding unnecessary toxicity in others. Additionally, the optimal duration of adjuvant ICI therapy remains an unresolved challenge. Current regimens, which are often administered over extended periods, necessitate careful consideration of the potential for cumulative toxicities over time. Balancing efficacy, minimizing adverse effects, and ensuring cost-effectiveness will be key priorities. Advances in molecular characterization have opened new opportunities to stratify patients based on tumor biology rather than solely on clinical features.

Cancer cells release cell-free DNA with tumor-specific molecular alterations into circulation. Multiple studies have documented the potential biomarker value of ctDNA for minimal residual disease detection and treatment response prediction in bladder cancer. Its short half-life in circulation (<2 h) makes it possible to use ctDNA for the real-time tracking of tumor burden following surgery and throughout oncological treatments [49]. However, the challenges of incorporating ctDNA into clinical decision making are its cost and that it is often inaccessible outside major academic centers. Additionally, prospective validation is still pending, making it difficult to establish standardized clinical applications [50].

Currently, ctDNA serves as an informative tool but does not alter standard clinical practice. NAC remains recommended regardless of ctDNA status, and its detection during neoadjuvant treatment does not lead to therapeutic changes beyond updated imaging. Similarly, in the adjuvant setting, high-risk patients continue to receive nivolumab after radical cystectomy, even if ctDNA is undetectable. Nevertheless, ctDNA is expected to play a more active role in future clinical decision making. Studies like ABACUS and IMvigor010 have demonstrated the correlation between ctDNA positivity and treatment response, highlighting that ctDNA clearance during therapy is associated with improved recurrence-free survival.

Building on these findings, ongoing trials including TOMBOLA, IMvigor011, and MODERN are evaluating whether ctDNA-guided therapy can enhance survival, reduce overtreatment, and improve quality of life in MIBC. These studies explore diverse strategies, from sequential ctDNA testing to identify patients who truly benefit from adjuvant therapy, to post-cystectomy monitoring aimed at optimizing immunotherapy administration. While serial ctDNA assessments could enhance risk stratification and personalize treatment, routine monthly testing may be impractical due to logistical and financial constraints [35]. Therefore, future efforts should prioritize the identification of early indicators of response or rapid ctDNA clearance—such as those suggested in exploratory analyses of IMvigor010—to enable more feasible and effective ctDNA-guided treatment strategies.

pCR following neoadjuvant therapy has also emerged as a key prognostic marker in MIBC, strongly correlated with improved survival outcomes. In patients achieving pCR, bladder preservation strategies have gained increasing interest as a means to avoid radical cystectomy and its associated morbidities, potentially improving quality of life [20]. Careful patient selection is critical, incorporating biomarkers, advanced imaging, and molecular profiling to identify suitable candidates for conservative management. Moreover, recent advances such as immunotherapy and ctDNA monitoring hold promise in refining perioperative treatment strategies. Given the complexity of decision making in this setting, a multidisciplinary approach is essential to tailor therapy, balancing oncologic control with functional outcomes. As research progresses, personalized treatment plans guided by robust clinical and molecular evidence will be pivotal to optimizing both survival and quality of life in bladder cancer patients.

Looking forward, several critical questions remain unanswered. First, we need to determine how novel peri-operative therapies will affect subsequent lines of treatment and the management of patients who ultimately develop metastatic disease. A major unmet need also concerns histological variants, for which clinical trials and therapeutic options are scarce. As in other tumours, such as colorectal cancer, the precise role of ctDNA in monitoring patients with localized MIBC must be clearly defined. Finally, studies evaluating the impact of these treatments on patients’ quality of life are urgently required.

Despite undertaking an exhaustive review of the literature and major conferences to summarize the most recent data on the peri-operative management of MIBC, our study has several limitations. First, we did not perform a formal risk-of-bias assessment for the studies included in this manuscript. Because we aimed to cover all relevant emerging agents in this setting, the evidence base we summarize inevitably inherits the limitations of the original studies—most notably, non-randomised designs, small sample sizes, and immature data. In addition, the field is evolving rapidly, with new results presented several times each year. Consequently, any evidence synthesis conducted at a single time point, such as ours, risks becoming outdated soon after publication.

## 5. Conclusions

Perioperative management of MIBC is shifting, with immunotherapy showing promising efficacy in neoadjuvant and adjuvant settings. While cisplatin-based NAC remains standard, ICIs may become central pending results from larger trials. Emerging biomarkers and molecular profiling support personalized treatment and response prediction, which will identify patients who may benefit from intensified or de-escalated strategies. Future research should validate these strategies and define optimal sequencing. A multidisciplinary approach is essential to balance oncologic control with quality of life in these patients.

## Figures and Tables

**Table 1:** Summary of Single-Agent Immunotherapy Neoadjuvant Clinical Trials in MIBC.

Trial (NCT)	Clinical Setting	Sample Size	Phase	Status	Experimental Arm	Primary Endpoint
NCT02736266 (PURE-01)	T2-4a N0M0	174	II	Completed	pembrolizumab	pCR
NCT03212651 (PANDORE)	T2-4a N0/xM0	41	II	Completed	pembrolizumab	pCR
NCT03319745	T2-4a N0M0	23	II	Completed	pembrolizumab	Incidence of adverse events
NCT02662309 (ABACUS)	T2-4a N0M0	96	II	Completed	atezolizumab	pCR
NCT04624399 (ABACUS 2)	T2-4a N0M0	52	II	Recruiting	atezolizumab	pCR changes in T cell subpopulations
NCT02451423	T2-4a N0M0	23	II	Completed	atezolizumab	pCR, Change in CD3+ T cell count (cells/μm^2^)
NCT00362713	T2-4a N0M0	12	I	Completed	ipilimumab	Safety
NCT03498196 (BL-AIR)	T2-4a N0M0	1	I–II	Terminated (low accrual)	avelumab	Change in T cell subpopulations
NCT05226117 (SURE 01)	T2-T4 N0M0	56	II	Unknown	sacituzumab govitecan	pCR

MIBC: muscle invasive bladder cancer; NCT: number of clinical trial at clinicaltrials.gov; pCR: pathological complete response.

**Table 2:** Summary of immune combination therapy neoadjuvant clinical trials in MIBC.

Trial (NCT)	Clinical Setting	Sample Size	Phase	Status	Experimental Arm	Primary Endpoint
NCT03387761 (NABUCCO)	T3-4N0M0, T1-T4a N13M0	54	Ib	Completed	nivolumab + ipilimumab	Feasibility
NCT03520491 (CA209-9DJ)	T2-4a N0M0	45	II	Recruiting	nivolumab + ipilimumab	Patients who proceed to surgery
NCT02845323	T2-4a N0M0	15	II	Active, not recruiting	nivolumab + urelumab	CD8+ T cell density at cystectomy
NCT03532451	T2-4a N0M0	43	I	Completed	nivolumab + lirilumab	Incidence of adverse events
NCT04209114	T2-4a N0M0	114	III	Completed	nivolumab + NKTR-214	pCR, EFS
NCT03472274 (DUTRENEO)	T2-4aN0M0, T2-4a N1M0	101	II	Completed	durvalumab + tremelimumab	Antitumor activity
NCT02812420	T2-4a N0M0	54	I	Active, not recruiting	durvalumab + tremelimumab	Safety and tolerability
NCT03234153 (NITIMIB)	T2-4aN0M0, T2-T4a N13M0	6	II	Terminated (low accrual)	durvalumab + tremelimumab	ORR
NCT03773666 (BLASST-2)	T2-4aN0M0	12	I	Completed	durvalumab + oleclumab	Patients proceeding to surgery without DLT
NCT04610671	T2-T4a, N0-N1, M0	21	I	Active, not recruiting	cretostimogene grenadenorepvec + nivolumab	Incidence of adverse events
NCT03534492 (NEODURVARIB)	T2-4a N0M0	29	II	Completed	durvalumab + olaparib	pCR

MIBC: muscle invasive bladder cancer; NCT: number of clinical trial at clinicaltrials.gov; pCR: pathological complete response; EFS: event-free survival; ORR: objective response rate; DLT: dose-limiting toxicity.

**Table 3:** Summary of chemo-immunotherapy combo neoadjuvant studies in MIBC.

Trial (NCT)	Clinical Setting	Sample Size	Phase	Status	Experimental Arm	Primary Endpoint
NCT03674424 (AURA)	T2-T4aN0-N2 M0	137	II	Completed	avelumab + gemcitbine/cisplatin	pCR
NCT03294304 (BLASST-1)	T2-T4a N0 M0	43	II	Completed	nivolumab + gemcitbine/cisplatin	pCR
NCT03773666 (BLASST-2)	T2-T4a N0 M0	12	I	Completed	durvalumab +/− oleclumab	Safety and tolerability
NCT04919512 (SUNRISE4)	T2-T4a N0 M0	163	II	Active, not recruiting	cetrelimab +/− gemcitabine (TAR200)	pCR
NCT03558087	T2-4a N0M0	76	II	Completed	nivolumab + gemcitabine/cisplatin	pCR
NCT02365766	T2-4a N0M0	83	I–II	Completed	pembrolizumab + gemcitabine/cisplatin	Incidence of adverse events PaIR
NCT02690558	T2-4a N0/x M0	39	II	Active, not recruiting	pembrolizumab + gemcitabine/cisplatin	Pathological downstaging to <pT2
NCT04383743	T2-4a N0M0	17	II	Active, not recruiting	pembrolizumab + aMVAC	pCR
NCT03549715 (NEMIO)	T2-4a N0-1 M0	121	I–II	Active, not recruiting	durvalumab + tremelimumab + MVAC	pCR Toxicity
NCT02989584	T2-4a N0/x M0	54	I–II	Active, not recruiting	atezolizumab + gemcitabine/cisplatin	Safety
NCT06341400	T2-4a N0M0	55	I–II	Recruiting	RC48-ADC disitamab-vedotin + toripalimab	pCR

MIBC: muscle invasive bladder cancer; NCT: number of clinical trial at clinicaltrials.gov; pCR: pathological complete response; MVAC: methotrexate, vinblastine, adriamycin, and cisplatin; aMVAC: accelerated MVAC.

**Table 4:** Description of adjuvant immunotherapy clinical trials.

	CheckMate274	IMvigor010	AMBASSADOR
Description	Phase III, multicentre, double-blind, randomized	Phase III, multicentre, open-label, randomized	Phase III, multicentre, open-label, randomized
Disease setting	1. MIUC after surgery 2. High risk of recurrence 3. With or without NAC	1. MIUC after surgery 2. High risk of recurrence	1. MIUC after surgery 2. High risk of recurrence 3. NAC treatment: cisplatin-treated
Drug	Randomization 1:1 Nivolumab (anti-PD1): n = 335 Placebo: n = 356	Randomization 1:1: Atezolizumab (antiPD-L1): n = 406 Placebo: n = 403	Randomization: 1:1 Pembrolizumab (anti-PD1): n = 354 Placebo: n = 348
Median Age (years)	65	66	68
Prior NAC treatment	308 (43%)	385 (46%)	447 (64%)
Presence of N+	335 (47%)	420 (52%)	350 (50%)
PD-L1 expression	≥1%: 282 (40%)	IC0-1: 417 (52%)	Score ≥ 10: 404 (58%)
Median follow up (months)	21.9	20	44.8
DFS	Improved: 20.8 months nivolumab vs. 10.8 months placebo HR: 0.7 (98% CI 0.55–0.9), *p* < 0.001	Not improved: 19.4 months atezolizumab vs. 16.6 months placebo. HR: 0.89 (95% CI 0.74–1.08), *p* = 0.24	Improved: 29.6 months pembrolizumab vs. 14.2 months placebo HR: 0.73 (95% CI 0.59–0.9), *p* = 0.003
OS	Improved *: HR:0.72 (95%CI, 0.59–0.89), *p* < 0.001	Not improved: HR: 0.85 (85% CI 0.66–1.09) *p* > 0.5	Not improved HR: 0.98 (95% CI 0.86–1.26), *p* > 0.5
PD1/PD-L1 status	PD-L1+ had DFS greater benefit: 74.5% with nivolumab vs. 55.7% with placebo. HR = 0.55; (98.72% CI: 0.35–0.85); *p* < 0.001.	ctDNA analysis showed prognostic and potential benefit in PD-L1+, but not significant	PD-L1+ had DFS greater benefit (36.9 vs. 21 months) [23]
ctDNA status	Not analyzed	Analyzed	Not analyzed
Limitations	1. No specification which groups does not benefit from adjuvant treatment. 2. ctDNA was not analyzed	1. Did not meet primary endpoints: DFS nor OS.	1. Did not meet OS endpoint 2. Substantial crossover from placebo to nivolumab (21%) 3. ctDNA was not analyzed
Key outcomes	1. FDA approval: MIUC at high risk of recurrence 2. EMA approval: MIUC at high risk of recurrence, expressing PD-L1 ≥ 1%. [23]	1. ctDNA showed prognostic and predictive value, better than PD-L1.	1. Adjuvant pembrolizumab demonstrated a significant DFS improvement, even greater in PD-L1+

MIUC: muscle-invasive urothelial cancer; NAC: neoadjuvant chemotherapy; N+: positive lymphatic nodes; DFS: disease-free survival; OS: overall survival; HR: hazard ratio; ctDNA: circulating tumor DNA; FDA: Food and Drug Administration; EMA: European Medicines Agency; PD-L1: programmed death-ligand 1 (PD-L1). * Based on Kaplan–Meier curves, as the necessary follow-up duration to assess patient outcomes has not yet been reached.

**Table 5:** Ongoing ctDNA-guided intervention in MIBC.

	IMvigor 011	TOMBOLA	MODERN
Start	2021	2020	2024
Disease setting	1. MIUC (>T2) and/or N+ 2. With or without NAC	1. MIUC (cT2-4a) 2. NAC	1. MIUC (>T2) and/or N+ after NAC 2. MIUC (>T3) and/or N+ without NAC and cisplatin-ineligible
Patients(n)	520	282	1190
Study type	Phase III, randomized, interventional	Phase III, non-randomized, national (Denmark)	Phase II/III, randomized, interventional
ctDNA method	Tumor informed, 16-plez NGS	Tumor informed, ddPCR	Tumor informed, ddPCR
Intervention	ctDNA +: randomized to adjuvant atezolizumab or no adjuvant therapy ctDNA-: ctDNA based surveillance	ctDNA +: adjuvant atezolizumab ctDNA-: ctDNA based surveillance	ctDNA+ (cohort A): randomized (a) adjuvant nivolumab (b) nivolumab + relatlimab (LAG-3 inhibitor) ctDNA- (cohort B): randomized (a) adjuvant nivolumab (b) ctDNA based surveillance
Primary endpoint	DFS	Response rate (defined as ctDNA clearance and absence of radiographic disease)	Cohort A: OS and ctDNA clearance Cohort B: DFS

ctDNA: circulating tumor DNA, MIBC: muscle-invasive bladder cancer MIUC: muscle-invasive urothelial cancer, NAC: neoadjuvant chemotherapy, DFS: disease-free survival, OS: overall survival; NGS: next generation sequencing; ddPCR: Droplet Digital Polymerase Chain Reaction.

**Table 6:** Perioperative Trials for Cisplatin-Eligible Patients: chemotherapy and immunotherapy.

	NIAGARA	KEYNOTE-866	ENERGIZE
Study design	Phase III, randomized, placebo-controlled		
Status (Estimated Primary Completion)	Completed	Ongoing (2025)	Ongoing (2027)
Patient population	1. cT2-T4a N0-1 M0, cisplatin-eligible 2. histologic subtypes, divergent differentiation 3. CrCl of ≥40 mL/min.	1. cT2-T4a N0-1 M0, cisplatin-eligible	1. cT2-T4a N0 M0, cisplatin-eligible 2. Cr Cl ≥ 50 mL/min 3. Excluded: prior chemotherapy, RT, surgery (other than TURB)
Immunotherapy	Durvalumab (anti-PD-L1)	Durvalumab (anti-PD-L1)	Nivolumab (anti-PD-1) ± Linrodostat (IDO1 inhibitor)
Treatment timing	NAC (GC) + durvalumab RC Adjuvant durvalumab	NAC (GC) + pembrolizumab RC Adjuvant pembrolizumab	Arm B: NAC(GC) + Nivolumab + placebo RC Adjuvant Nivolumab + placebo; Arm C: NAC (GC) + Nivolumab + linrodostat RC adjuvant Nivolumab + linrodostat.
Control arm	NAC (GC)+ placebo RC Adjuvant placebo	NAC (GC)+ placebo RC Adjuvant placebo	Arm A: NAC(GC) alone
Primary endpoint	EFS, pCR	EFS, pCR	pCR EFS (arms C vs. A; B vs. A)
Secondary endpoints	OS, downstaging, safety, tolerability	OS, DFS, pathologic downstaging, safety, tolerability	OS, safety, tolerability
Biomarker integration	Exploratory: PD-L1, mRNA panels, TMB	Exploratory: PD-L1, mutations-FGFR	Exploratory: PD-L1, IDO1 expression, TMB
Unique features	Potential new treatment for cisplatin-eligible MIBC	Large global trial with pembrolizumab	Adds IDO1 inhibition to PD-1 blockade, targeting tumor immune evasion

CrCl: creatinine clearance, RT: radiotherapy, TURB: transurethral resection of the bladder, NAC: neoadjuvant chemotherapy, GC: gemcitabine + cisplatin, RC: radical cystectomy, EFS: event-free survival, pCR: pathological complete response, DFS: disease-free survival, IDO1: Indoleamine 2, 3-dioxygenase 1; TMB: tumour mutational burden; OS: overall survival; PD-L1: programmed death-ligand 1 (PD-L1); mRNA: messenger ribonucleic acid; FGFR: Fibroblast Growth Factor Receptor.

**Table 7:** Summary of perioperative studies in MIBC.

Trial (NCT)	Clinical Setting	Sample Size	Phase	Status	Experimental Arm	Primary Endpoint
NCT01812369 (VESPER)	T2-4a N0-Nx M0	500	III	Completed	gemcitabine/cisplatin/ddMVAC	PFS
NCT02177695 (COXEN)		237	II	Completed	gemcitabine/cisplatin/ddMVAC	Predictive value of COXEN score for pCR
NCT03732677 (NIAGARA)	T2-4a N0M0	1063	III	Active, not recruiting	durvalumab + gemcitabine/cisplatin	pCR EFS
NCT05535218 (SURE02)	T2-T3bN0M0	48	II	Active, not recruiting	pembrolizumab+ sacituzumab govitecan	pCR
NCT03661320 (ENERGIZE)	T2-4a N0M0	861	III	Active, not recruiting	nivolumab + gemcitabine/cisplatin +/− BMS-986205	pCR EFS
NCT03924895 (KEYNOTE-905/EV-303)	cT2-T4aN0M0/T1-T4a N1M0	595	III	Active, not recruiting	pembrolizumab+/− enfortumab vedotin	pCR EFS
NCT03924856 (KEYNOTE-866)	T2-T4aN0M0/T1-T4a N1M0	907	III	Active, not recruiting	pembrolizumab+ gemcitabine/cisplatin	pCR EFS
NCT03406650 (SAKK 06/17)	T2-T4a N0-1 M0	61	II	Active, not recruiting	durvalumab + gemcitabine/cisplatin	EFS
NCT04876313 (NURE-Combo)	T2-T4a N0M0	29	II	Recruiting	nivolumab+ nab-paclitaxel	pCR
NCT04960709 (VOLGA)	T2-T4aN0-1M0 T1N1M0	712	III	Active, not recruiting	durvaluamb + enfortumab vedotin +/− tremelimumab	Safety and tolerability, pCR, EFS
NCT05297552 (RC48-C017)	cT2-T4a N0-1 M0	40	II	Recruiting	disitamab vedotin + toripalimab	pCR
NCT04209114 (PIVOT IO 009)	T2-T4aN0M0 T1-T4a N1M0	114	III	Completed	nivolumab + bempegaldesleukin	pCR EFS

MIBC: muscle invasive bladder cancer; NCT: number of clinical trial at clinicaltrials.gov; PFS: progression-free survival; pCR: pathological complete response; EFS: event-free survival; ddMVAC: dose-dense MVAC.

## Abbreviations

The following abbreviations are used in this manuscript:

AC	Adjuvant Chemotherapy
ADC	Antibody–Drug Conjugate
AI	Artificial intelligence
BCG	Bacillus Calmette–Guérin
CISCA	Cisplatin + Cyclophosphamid + Adriamycin
CM	Cisplatin + Methotrexate
CMV	Cisplatin + Methotrexate + Vinblastine
ctDNA	circulating tumor DNA
CTLA-4	Cytotoxic T-lymphocyte–associated protein 4
dd-MVAC	dose-dense Methotrexate, Vinblastine, Doxorubicin, and Cisplatin
DFS	Disease-Free Survival
DV	Disitamab Vedotin
EFS	Event-Free Survival
EMA	European Medicines Agency
EV	Enfortumab Vedotin
FDA	Food And Drug Administration
HR	Hazard Ratio
MIUC	Muscle-Invasive UC
MVAC	Methotrexate + Vinblastine + Adriamycin + Cisplatin
NAC	Neoadjuvant chemotherapy
NMIBC	Non–Muscle-Invasive Bladder Cancer
OS	Overall Survival
PARP	Poly (ADP-ribose) polymerase
pCR	Pathological Complete Response
PD-1	Programmed death-1
PD-L1	Programmed Death-Ligand 1
PFS	Progression-Free Survival
RCT	Randomized Controlled Trials
RFS	Recurrence-Free Survival
TGF-β	Transforming Growth Factor beta
TMB	Tumor mutational burden
TURBT	Transurethral Resection of Bladder Tumor
TROP2	Trophoblast cell-surface antigen 2
UC	Urothelial Carcinoma
